# Texas Wool, Oil, and Beef

**Published:** 1877-12

**Authors:** 


### *TEXAS WOOL, OIL AND BEEF.*

It seems that California is losing while Texas is gaining prestige in wool growing, and by close calculations five years will show the immense acreage of Texas to be supporting more cattle and sheep than the whole country besides. No wonder, then, at the half million emigration to Texas yearly.

With rail facilities rapidly increasing, cheap lands, no feeding of stock in winter, no expensive farm buildings, it appears to be the most attractive State for the small or large farmer. Some drawbacks as regards water must be met by pumps, and windmills, and systematic irrigating ditches. The fostering State laws have induced capitalists to build several long canals to supply water to farmers, to enable them to raise two crops of corn a year, on the same ground. Prices of lands are doubling yearly, near railroads; the investor cannot, therefore, lose, and the exercise of judgment will enable him to realize a large profit.

Our old friend, Dr. G. Duryee, 176 Broadway, New York, whom we have known for years, and whose word can be relied on, can furnish statistics of tho profits of stock raising, procured from old ranchmen in Texas. His experience as a practical consulting chemist has been extensive. He attests the wonderful preservative properties of xantogenate of sodium for purifying air-tight cans and vessels employed in the shipment of beef. Zoller places this salt among the very best preservative agents, and its cheapness will allow of its being used in shipping San Antonio steaks, sweet and juicy, from Texas to New York and Europe. The estimated annual wool-product of the United States, is two hundred million pounds, worth about fifty million dollars. Texas and California each yield one-quarter of the whole product. Cotton-seed oil is worth nearly as much as linseed. Five million gallons can be made from seed now wasted in Texas annually. In this, chemistry has stepped in with new processes and surer modes.

Angora goats, whose fleece is worth from one to three dollars a pound, seem to thrive well in Texas. If sheep-grazers would use business common-sense in selecting fine wool breeds, Texas wool could soon bring forty cents instead of twenty, with no extra expense aside from first outlay. A thousand common sheep and six hundred and forty acres of land, both costing about three thousand dollars, clear twenty thousand dollars in five years. When sheep-ranches can be got so easily, there would seem to be no excuse for plucky young merchants complaining of slow trade and poor collections. Suppose we suggest to such persons that they trade their goods for land, and start, provided always they can live one year without income. We don't mean this as advice, but throw out the hint for the consideration of all who are not "getting on in the world."

Let us offer another hint. It has always appeared to us that wool should at least be cleaned and carded before shipment, and if burred wool could be treated in Texas as it is in New England, the value would be about fifty cents instead of ten.

Now for hint number three. There is all over Texas a rank-growing grass, which can be made into splendid paper. Why cannot this be gathered, and the dirty hospital-rags now used by paper-makers be burned ?

Hint number four. Texas beef, boiled and put in cans similar to sardine cans, with the refined cotton seed oil, which is largely supplanting olive oil, keeps perfeetly, and will sell at a large profit all over the world.

Hint number five. We don't care to praise "oleomargarine," and yet we notice that consumers are unable to tell "which is which," when this substance and choice butter are placed side by side. Chemically, they are identical, being made of animal fat and sweet milk. Oleomargarine is sure to be largely used, and Texas is the best country in the world in which to manufacture it, because there sweet milk and sweet suet cost next to nothing.

These hints are sufficient for this month. For the young man who would better his condition, Texas would seem to be the best spot on the continent. She is destined to be an enormous producer of articles essential to human needs. We might safely modify Mr. Greeley's advice, and say, "Young man, go to Texas;" and when you go, take your family and make a home there, and aid in the re-arrangement of society.

